# A detailed explanation and graphical representation of the Blinder-Oaxaca decomposition method with its application in health inequalities

**DOI:** 10.1186/s12982-021-00100-9

**Published:** 2021-08-06

**Authors:** Ebrahim Rahimi, Seyed Saeed Hashemi Nazari

**Affiliations:** 1 Department of Public Health, Mamasani Higher Education Complex for Health, Shiraz University of Medical Sciences, Shiraz, Iran; 2 Prevention of Cardiovascular Disease Research Center, Department of Epidemiology, School of Public Health and Safety, Shahid Beheshti University of Medical Sciences, Velenjak St., Chamran Highway, Tehran, Iran

**Keywords:** Health Inequality, Decomposition methods, Contributing factors

## Abstract

This paper introduces the Blinder-Oaxaca decomposition method to be applied in explaining inequality in health outcome across any two groups. In order to understand every aspect of the inequality, multiple regression model can be used in a way to decompose the inequality into contributing factors. The method can therefore be indicated to what extent of the difference in mean predicted outcome between two groups is due to differences in the levels of observable characteristics (acceptable and fair). Assuming the identical characteristics in the two groups, the remaining inequality can be due to differential effects of the characteristics, maybe discrimination, and unobserved factors that not included in the model. Thus, using the decomposition methods can identify the contribution of each particular factor in moderating the current inequality. Accordingly, more detailed information can be provided for policy-makers, especially concerning modifiable factors. The method is theoretically described in detail and schematically presented. In the following, some criticisms of the model are reviewed, and several statistical commands are represented for performing the method, as well. Furthermore, the application of it in the health inequality with an applied example is presented.

## Introduction

Inequality is one of the most obvious facts perceived in human life, referring to differences affecting the individual way of life. In spite of this simple meaning, inequality involves complexities hindering a consensus on its definition. Thus, inequality has been subject to numerous research projects. This concept is generally defined according to different needs and conditions of individuals. It is therefore related to the conditions and characteristics of recipients rather than providers of special services [1].

In some cases, inequality is used interchangeably with inequity. Any judgment to what extent inequality should be considered inequity will depend on the unfairness [2].

Discrimination, alongside the sense of inequity, refers to a situation in which belonging to a particular group sets the ground for preference or non-preference of that group [3]. In spite of identical capabilities and characteristics, the individuals in each group receive different benefits and services, related to the specific position of that group compared to others.

In the realm of public healthcare, inequality is a term referring to differences, dissimilarities and disparities visible in the health status of individuals or groups, which is indirectly applied as a tool to measure health inequity. In other words, one instance of inequity is the systematic and avoidable inequalities in groups with identical characteristics [4].

Despite the overall improvement in global health and hygiene, inequality has escalated over recent centuries. This issue can be mitigated inevitably through identification of the contributing factors. However, achievement of equity in the health sector is known as a major challenge facing the relevant authorities (2, 5). Although a great deal of investigation has been conducted on disparities in the health sector and medical sciences, very little research has focused on how to reduce it [5]. The first step in the formulation, design and implementation of effective interventions to reduce health inequalities is an investigation into the contributing factors and causes [6].

To that end, and in order to understand every aspect of inequality, multiple regression model can be used in a way to decompose inequality into its components. In 1973, Blinder [7] and Oaxaca [8] proposed a new method to examine the factors associated with racial/gender wage inequality and discrimination in the labor market. This method can be applied to explain inequalities in health outcome across any two groups.

The aim of the method is to explain how much of the difference in mean outcome between two groups is due to group differences in the levels of observed characteristics (acceptable and fair) and how much is due to discrimination, but may also result from the differential effect of the observed characteristics (group difference in the magnitude of regression coefficients) as well as other unknown associated factors. In fact, existence of inequality despite identical individual characteristics can be rooted in unknown factors that affect the outcomes. Thus, the difference in the outcome can be adjusted by mitigating the difference in the level of associated factors across comparison groups. The rest will concern unmeasurable, unobserved factors [9, 10]. Therefore, this method can be employed to identify the contribution of each factor into inequality.

## Main text

### Blinder-Oaxaca (B-O) decomposition method

Sometimes it is essential to decompose the mean difference in a specific continuous outcome between 2 groups (Group 1 and Group 2) to determine the factors contributing to that difference. For this purpose, multiple regression model can be employed. In terms of statistical measures, this particular decomposition method can be considered a combination of t-test and multiple regression models. Assuming that the outcome value (*Y*) is explained by K variables (*x*_1_, ….*x*_*k*_) in the linear regression model, the mean predicted outcome for group *g* (1 and 2) can be expressed as follows:

$$\overline{Y}^{g} = \beta_{0}^{g} + \mathop \sum \limits_{j = 1}^{k} \beta_{j}^{g} \overline{x}_{j}^{g}$$

where $\overline{x}_{j}$ is the mean value of each predictor and $\beta$ is the estimated regression coefficient.

Thus, the mean difference in outcome between the 2 groups (1 and 2) is as follows:

1 $$\Delta \overline{Y} = \left( {\beta_{0}^{1} - \beta_{0}^{2} } \right) + \mathop \sum \limits_{j = 1}^{k} (\beta_{j}^{1} \overline{x}_{j}^{1} - \beta_{j}^{2} \overline{x}_{j}^{2} )$$

The mean difference of outcome is the sum of the effects of different components, including: (1) Average difference between the level of each observable variable ($x_{j}$); (2) differential effects ($\beta_{j}$) of these variables in the 2 comparison groups, and (3) basic difference which includes the effect of unknown variables that are not included in the model. One question worth asking is “How large is the contribution of each of model components to this difference?".

To answer this question, the levels of explanatory variables and regression coefficients in the two groups are alternately assumed identical to achieve the net effect of each component. In fact, a counterfactual approach is adopted to replace the coefficients and the variables levels of the equation for one group to corresponding values for the other group (reference). Accordingly, the expected change in a group mean outcome is obtained when this group gets the predictor values and regression coefficients of the reference. In this procedure, the contribution of each component can be estimated [9, 11].

If Group 1 (or its outcome) is selected as the reference, the expected change in predictors level and regression coefficients of Group 2 and subsequently the change in its outcome will be considered.

The equation exclusive to Group 1 can be reformulated from the perspective of Group 2 as follows:

$$\begin{aligned} \overline{Y}^{1} & = \beta_{0}^{1} + \mathop \sum \limits_{j = 1}^{k} \beta_{j}^{1} \overline{x}_{j}^{1} \\ & = \beta_{0}^{1} + \mathop \sum \limits_{j = 1}^{k} \left[ {\beta_{j}^{2} + (\beta_{j}^{1} - \beta_{j}^{2} } \right)]\mathop \sum \limits_{j = 1}^{k} \left[ {\overline{x}_{j}^{2} + \left( {\overline{x}_{j}^{1} - \overline{x}_{j}^{2} } \right)} \right] \\ & = \beta_{0}^{1} + \mathop \sum \limits_{j = 1}^{k} \beta_{j}^{2} \overline{x}_{j}^{2} + \mathop \sum \limits_{j = 1}^{k} \beta_{j}^{2} \left( {\overline{x}_{j}^{1} - \overline{x}_{j}^{2} } \right) + \mathop \sum \limits_{j = 1}^{k} \overline{x}_{j}^{2} (\beta_{j}^{1} - \beta_{j}^{2} ) + \mathop \sum \limits_{j = 1}^{k} \left( {\overline{x}_{j}^{1} - \overline{x}_{j}^{2} } \right)\left( {\beta_{j}^{1} - \beta_{j}^{2} } \right) \\ \end{aligned}$$

The above equation involves $\beta_{j}^{1} = \beta_{j}^{2} + (\beta_{j}^{1} - \beta_{j}^{2} )$ and $\overline{x}_{j}^{1} = \overline{x}_{j}^{2} + \left( {\overline{x}_{j}^{1} - \overline{x}_{j}^{2} } \right)$, which can be replaced in Eq. 1 to decompose the mean difference in outcome into 4 components as follows:
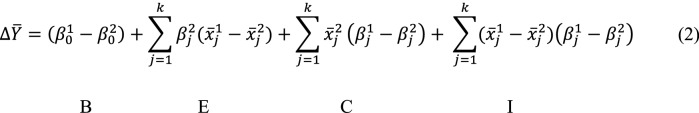


The decomposition shown in this equation is formulated from the perspective of Group 2, when Group 1 is selected as the reference.

Accordingly, the predicted difference (D) can be decomposed into 4 components (B, E, C and I); in other words, the contribution of each component in the difference can be estimated:

1. The first component (B) is attributed to basic differences. It includes the effects of unobservable variables not taken into account (i.e. not included in the model).
2. The second component (E) indicates change in group 2’s mean predicted outcome when it meets the group 1 (Reference)’s covariates level:
  $$\mathop \sum \limits_{j = 1}^{k} \beta_{j}^{2} (\overline{x}_{j}^{1} - \overline{x}_{j}^{2} ) = \mathop \sum \limits_{j = 1}^{K} \left( {\beta_{j}^{2} \overline{x}_{j}^{1} - \beta_{j}^{2} \overline{x}_{j}^{2} } \right)$$
  In other words, a portion of the difference (D) that explained by group differences in the level of observable explanatory variables (explained component). This portion is known as “endowments effect”.
3. The third component(C) is a part of the difference represents a change in group 2’s mean predicted outcome when that group meets the regression coefficients of the other group:
  $$\mathop \sum \limits_{j = 1}^{k} \overline{x}_{j}^{2} \left( {\beta_{j}^{1} - \beta_{j}^{2} } \right) = \mathop \sum \limits_{j = 1}^{K} \left( {\beta_{j}^{1} \overline{x}_{j}^{2} - \beta_{j}^{2} \overline{x}_{j}^{2} } \right)$$
  It involves a portion of the difference (D) caused by the differential effect of the observable variables on outcome across the 2 comparison groups. It cannot be explained by the level of observable explanatory variables (unexplained component). This portion of the difference is known as “coefficients effect”.
4. The fourth component (I) involves an interaction due to simultaneous effect of differences in endowments and coefficients [11, 12].

**Fig. 1:**
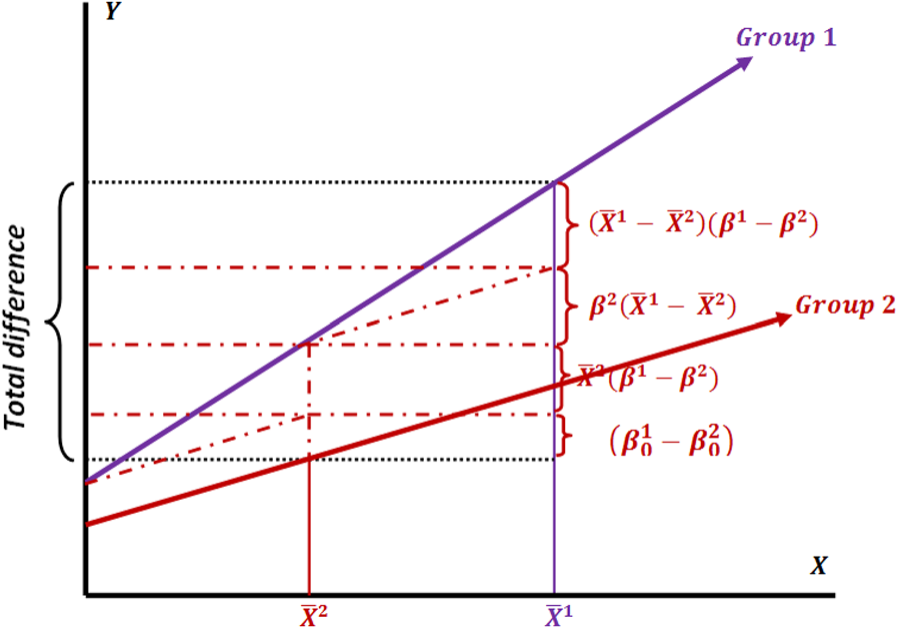
Decomposition of the group difference in mean predicted outcome (the interaction model) by selecting Group 1 as the reference (from the perspective of Group 2)

Figure 1 schematically displays the decomposition of group difference in mean predicted outcome from the perspective of Group 2, when Group 1 has been considered a reference (Eq. 2)

Similarly, when Group 2 is selected as the reference, the expected change in mean predicted outcome can be expressed from the perspective of Group 1 as follows:
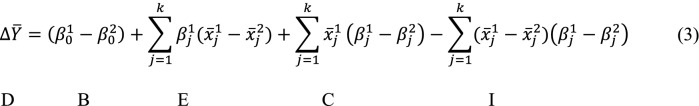


To achieve the above Eq. 3, the covariates level and regression coefficients of Group 2 are reformulated from the perspective of Group 1 as follows

$${\upbeta }_{{\text{j}}}^{2} \, = \,\upbeta _{{\text{j}}}^{1} - \left( {{\upbeta }_{{\text{j}}}^{1} - {\upbeta }_{{\text{j}}}^{2} } \right)$$

$$\overline{x}_{j}^{2} = \overline{x}_{j}^{1} - \left( {\overline{x}_{j}^{1} - \overline{x}_{j}^{2} } \right)$$

And then replace their corresponding values in the Eq. 1. Thus, the difference (D) in Eq. 3 can be decomposed into 4 partitions (B, E, C and I): The first component (B) and fourth component (I) are related to the same factors expressed in the following Eq. 2. The second component (E), however, measures expected change in group 1’s mean predicted outcome if this group has the group 2(Reference)’s covariates level:

$$\mathop \sum \limits_{j = 1}^{k} \beta_{j}^{1} (\overline{x}_{j}^{1} - \overline{x}_{j}^{2} ) = \mathop \sum \limits_{j = 1}^{K} \left( {\beta_{j}^{1} \overline{x}_{j}^{1} - \beta_{j}^{1} \overline{x}_{j}^{2} } \right)$$

The third component (C) is similarly a part of difference measures the expected change in group 1’s mean predicted outcome when this group meets the regression coefficients of Group 2:

$$\mathop \sum \limits_{j = 1}^{k} \overline{x}_{j}^{1} \left( {\beta_{j}^{1} - \beta_{j}^{2} } \right) = \mathop \sum \limits_{j = 1}^{K} \left( {\beta_{j}^{1} \overline{x}_{j}^{1} - \beta_{j}^{2} \overline{x}_{j}^{1} } \right)$$

**Fig. 2:**
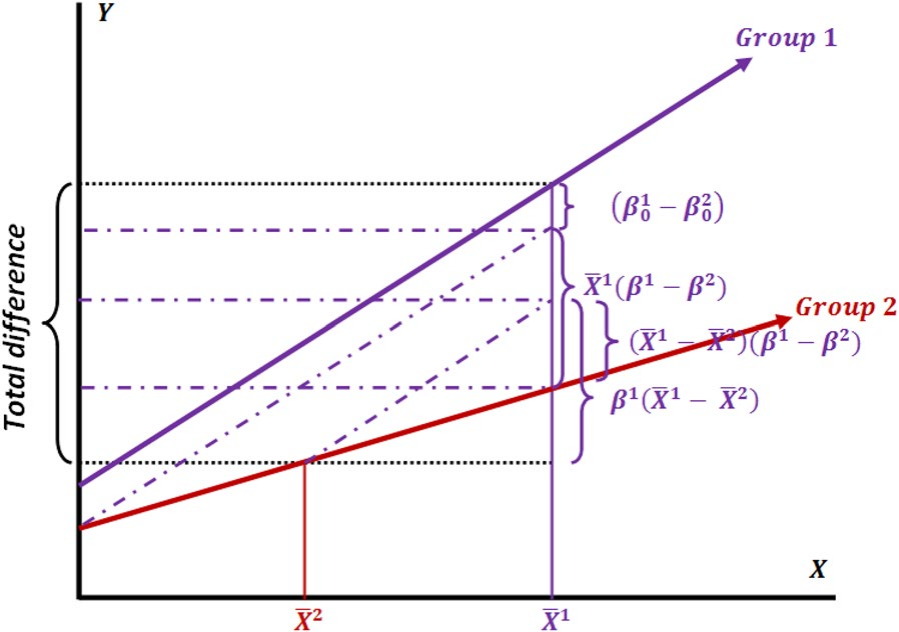
Decomposition of difference (the interaction model) by selecting Group 2 as the reference (from the perspective of Group 1)

Figure 2 schematically depicts the decomposition conditions where Group 2 has been selected as a reference.

In Eqs. 2 and 3 (Figs. 1 and 2) the first component (B), $\left( {\beta_{0}^{1} - \beta_{0}^{2} } \right)$, denotes to the differences between two groups that cannot be explained by the observed covariates (X). In fact, this difference is due to unobserved variables. On the other hand, the coefficient component (part C) is also unexplained by those differences. Then, we can combine these two components (B and C) into unexplained part (U), yielding the three-fold decomposition,

4 $$\Delta \overline{Y} = \mathop \sum \limits_{j = 1}^{k} \beta_{j}^{2} (\overline{x}_{j}^{1} - \overline{x}_{j}^{2} ) + \mathop \sum \limits_{j = 1}^{k} \overline{x}_{j}^{2} \left( {\beta_{j}^{1} - \beta_{j}^{2} } \right) + \mathop \sum \limits_{j = 1}^{k} (\overline{x}_{j}^{1} - \overline{x}_{j}^{2} )\left( {\beta_{j}^{1} - \beta_{j}^{2} } \right)$$

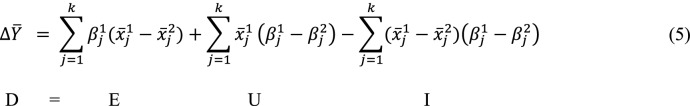


In other words, if we assume that there are no relevant unobservable explanatory variables, the total unexplained part (U) in the Eqs. 4 and 5 will be equal to the C component in the Eqs. 2 and 3.

In this approach, the difference in mean predicted outcome (D) contains three components (E, U and I): The first component (E) is explained by the difference in the level of the covariates, the second component (U) arises from the differential effect of all those covariates (the unexplained part), and the third component (I) involves an interaction caused by the simultaneous group differences in the covariates level and their coefficients.

Up to now we had postulated that one of the groups 1 or 2 has the best achievable outcome and the other group should reach to this outcome. Another approach is that we suppose that there is a nondiscriminatory condition (marked by a nondiscriminatory vector of coefficients) that both groups should reach to this condition. Therefore, this approach requires the definition of nondiscriminatory conditions or reference coefficients. Sometimes even this nondiscriminatory condition can be the situation of one of the comparison groups (which we can call it reference coefficient).

Suppose $\beta^{*}$ is the nondiscriminatory condition or reference coefficient, the overall equation for decomposition of $\Delta \overline{Y}$ will be:.
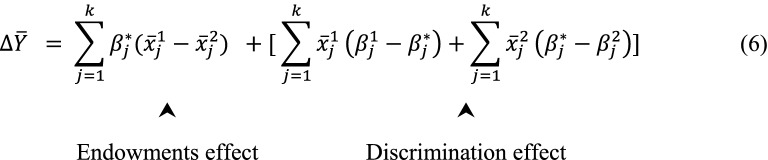


In this way the outcome difference has been decomposed into two components (two-fold decomposition). The first component is the part of the group difference that is explained by the differences in the levels of observed characteristics. This is also called “endowments effect”. The second component refers to the part of the gap that is due to differences of ${\upbeta }$ s with the non-discriminating ${\upbeta }^{*}$. It also catches differences in level of unobservable variables and also their differential (discriminating) effects. This component determines the unexplained portion of the disparity. If all the unobserved covariates were in the model and measured, it comprised just the difference of ${\upbeta }$ *s* with the non-discriminating ${\upbeta }^{*}$. This portion is sometimes considered as “discrimination effects”.

${\upbeta }^{*}$ is always between ${\upbeta }^{{1}}$ and ${\upbeta }^{2}$ , or equal to both or one of them. Then, we have ${\upbeta }^{{1}} \ge {\upbeta }^{*} \ge {\upbeta }^{{2}}$ or ${\upbeta }^{{1}} \le {\upbeta }^{*} \le {\upbeta }^{{2}}$.

If ${\upbeta }^{{1}} > {\upbeta }^{{*}} > {\upbeta}^{{2}}$, we have positive discrimination "in favor of" group 1 and negative discrimination “against" group 2 and if ${\upbeta }^{{1}} { < \upbeta }^{*} { < \upbeta }^{{2}}$, then we have positive discrimination in "in favor of" group 2 and negative discrimination "against" Group 1.

There is also a case that one of the two groups experiences discrimination and the non-discriminating ${\upbeta }^{*}$ will simply be the coefficients from the other group. In such case, ${\upbeta }^{{1}} { = \upbeta }^{*} {> \upbeta }^{{2}}$ or ${\upbeta }^{{1}} > {\upbeta }^{*} { = \upbeta }^{{2}}$. If we replace ${\upbeta }^{*}$ with ${\upbeta }^{1}$ in the Eq. 6, we reach to the Eq. 7 and if we replace ${\upbeta }^{*}$ with ${\upbeta }^{2}$ we reach to the Eq. 8

7 $$\Delta \overline{Y} = \mathop \sum \limits_{j = 1}^{k} \beta_{j}^{1} (\overline{x}_{j}^{1} - \overline{x}_{j}^{2} ) + \mathop \sum \limits_{j = 1}^{k} \overline{x}_{j}^{2} \left( {\beta_{j}^{1} - \beta_{j}^{2} } \right)$$

8
.

Therefore, we have a twofold decomposition of the difference in mean predicted outcome (D):

1. The Unexplained component (Uc): This is exactly similar to the U part of the three-fold decomposition (Eq. 4 and 5). It arises from the differential effect of observable variables and also differential effect (${\upbeta }$) and level of unobservable variables. This determines the unexplained portion of the gap.
2. The Explained component (Ec): This part is the combination of E and I parts of the three-fold decomposition (Eqs. 4 and 5). Although this component is called the explained component in two-fold decomposition in many texts but some part of it (the interaction part) is in fact the simultaneous difference of coefficients and covariates level in both groups. Hence, if somebody wants the crude explained component, three folds’ decomposition can provide this crude explained part [11, 13].

Therefore, Eqs. 7 and 8 can be considered a specific form of Eqs. 4 and 5, where components *E* and *I* have been integrated. Thus:

$$\mathop \sum \limits_{j = 1}^{k} \beta_{j}^{2} (\overline{x}_{j}^{1} - \overline{x}_{j}^{2} ) + \mathop \sum \limits_{j = 1}^{k} (\overline{x}_{j}^{1} - \overline{x}_{j}^{2} )\left( {\beta_{j}^{1} - \beta_{j}^{2} } \right) = \mathop \sum \limits_{j = 1}^{k} \beta_{j}^{1} (\overline{x}_{j}^{1} - \overline{x}_{j}^{2} )$$

and

$$\mathop \sum \limits_{j = 1}^{k} \beta_{j}^{1} (\overline{x}_{j}^{1} - \overline{x}_{j}^{2} ) - \mathop \sum \limits_{j = 1}^{k} (\overline{x}_{j}^{1} - \overline{x}_{j}^{2} )\left( {\beta_{j}^{1} - \beta_{j}^{2} } \right) = \mathop \sum \limits_{j = 1}^{k} \beta_{j}^{2} (\overline{x}_{j}^{1} - \overline{x}_{j}^{2} )$$

**Fig. 3:**
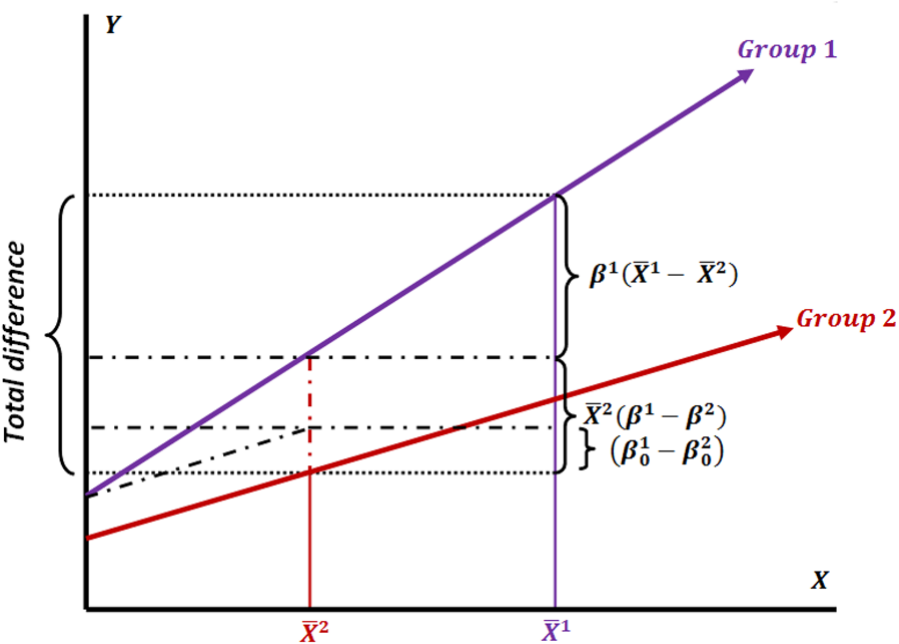
Decomposition of outcome difference using the group 1 coefficients as the reference

**Fig. 4:**
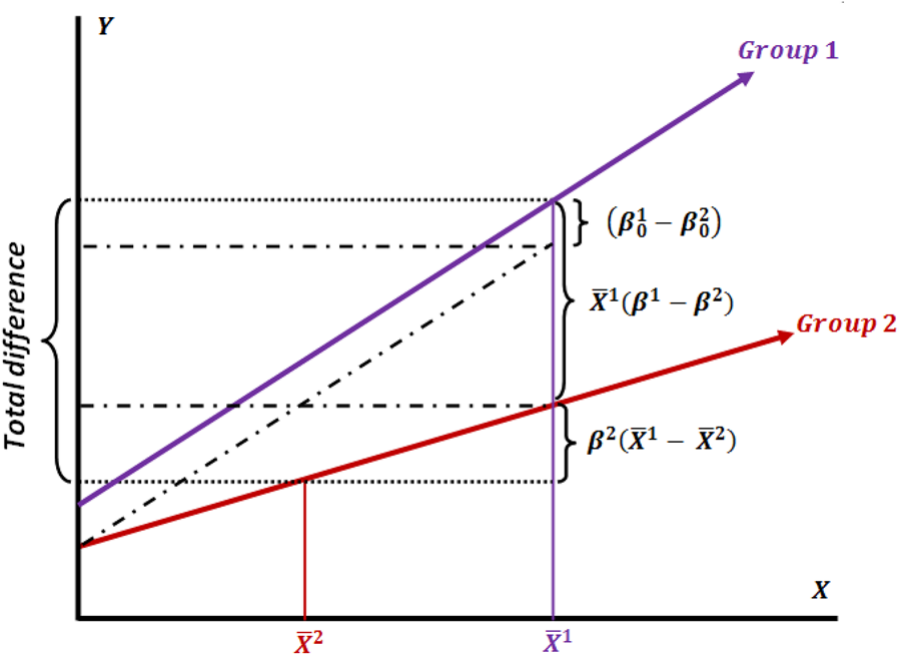
Decomposition of outcome difference using the group 2 coefficients as the reference

Figures 3 and 4 schematically demonstrate the decomposition conditions where the group 1 and group 2 coefficients has been selected as a reference, respectively.

It is not clear which regression coefficient should be selected as a reference (Eqs. 7and 8). This is known as "index problem” [9, 14–18]. Reimers [19] suggests using the average regression coefficients over both groups ($\frac{{\upbeta _i^1 + \upbeta _i^2}}{2}$), while Cotton [15] expresses the sum of coefficients weighted by each group size ($\frac{{n^{1} {\upbeta }_{{\text{i}}}^{1} + n^{2} {\upbeta }_{{\text{i}}}^{2} }}{{\text{N}}}$) for ${\upbeta }_{{\text{j}}}^{*}$. In this regard, Neumark proposes the use of regression coefficients from a pooled model over both groups as an estimate for nondiscriminatory conditions [16, 17].

### Nonlinear extension of B-O decomposition method

Although the primary application of the proposed B-O decomposition is based on the linear regression model, several researchers, including Yun and Fairlie, have proposed a nonlinear version of decomposition [14, 20–22], which has been widely used in the decomposition of inequalities in the health sector [23–27].

As mentioned, the original B-O decomposition of the 2-group disparity in the average value of the response variable, Y, can be expressed as:

$$\Delta \overline{Y} = [\beta^{1} \left( {\overline{X}^{1} - \overline{X}^{2} } \right)\left] + \right[\overline{X}^{2} \left( {\beta^{1} - \beta^{2} } \right)]$$

where $\overline{X}$ is a row vector of average values of the explanatory variables and $\beta$ is a vector of coefficient estimates for each group 1 and 2. In this case, the coefficient estimates of group 1, $\beta^{1}$, have been assumed to be as the reference. According to Fairlie[28], the decomposition for a nonlinear equation, $Y = F\left( {X\beta } \right)$ can be expressed as follows: 
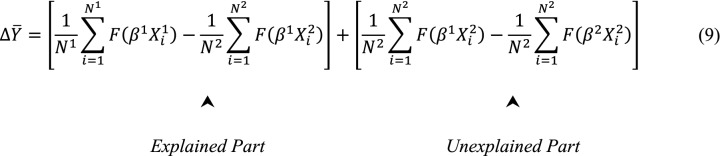
 where *N*^*g*^ is the sample size for group g (1 or 2), and $\Delta {\overline{\text{Y}}}$ represents the difference in "mean predicted probability of outcome" between two groups with N^1^ and N^2^ individuals. This alternative expression for the decomposition is used because in non-linear transformations of Y, $\overline{Y}$ does not necessarily equal $F\left( {\overline{X}\beta } \right)$. The original B-O decomposition is a special case of Eq. 9 in which $F\left( {X_{i} \beta } \right) = X_{i} \beta$ [28].

Similarly, another expression for the decomposition is:

10 $$\Delta \overline{Y} = \left[ {\frac{1}{{N^{1} }}\mathop \sum \limits_{i = 1}^{{N^{1} }} F\left( {\beta^{2} X_{i}^{1} } \right) - \frac{1}{{N^{2} }}\mathop \sum \limits_{i = 1}^{{N^{2} }} F\left( {\beta^{2} X_{i}^{2} } \right)} \right] + \left[ {\frac{1}{{N^{1} }}\mathop \sum \limits_{i = 1}^{{N^{1} }} F\left( {\beta^{1} X_{i}^{1} } \right) - { }\frac{1}{{N^{1} }}\mathop \sum \limits_{i = 1}^{{N^{1} }} F\left( {\beta^{2} X_{i}^{1} } \right)} \right]$$

where the vector of coefficient estimates for group 2 is used as the reference.

### Detailed decomposition

In the detailed decomposition one can determine the relative contribution of each factor (X variables) to each one of explained and unexplained components. This can be achieved by sequentially substituting variables levels/coefficients of one group with those of another group while keeping other variables in the model constant.

Using linear regression based decomposition; the detailed decomposition is not a complicated task because each component is obtained simply by summing over the contribution of each predictor to the each component. In nonlinear method, however, performing the detailed decomposition is not as straightforward. In other words, the application of the original (linear) method to nonlinear decomposition models has some conceptual problems that affect the results [28–31]. The first problem is known as “identification problem”, that is, for nominal (categorical) variables, as the predictors, the decomposition estimates depend on the choice of the base (omitted) category. One solution, proposed by Yun [30], is computing normalized effects. It is equivalent to averaging the coefficients effects of a set of dummy variables while changing the reference groups.

Another problem is “path dependency”. Unlike linear models, nonlinear decomposition is sensitive also to the order of variables being included into the decomposition process (path dependency) [22, 28, 29, 32]. One solution to this issue has been suggested by Fairlie, which involves randomly ordering the variables across replications of the decomposition. This procedure requires one-to-one matching of individuals from the 2 comparing groups, thus there should be equal number of individuals in both groups $\left( {{\text{N}}^{1} = {\text{N}}^{2} } \right){ }$. Otherwise (which is usually the case), a random subsample of the majority group 1 (which is usually equal to the sample size of minority group 2) will be selected and then matched according to the predicted probability for the response variable of each person. In fact, the individual observations in each group will be separately arranged based on predicted probability and then matched according to ranking. This procedure will match the individual characteristics in both groups. Thus, the matched observations (one-to-one) will determine the contribution of each factor to the outcome difference. Thus, the multiple sub-samples (e.g. 100 or 1000 times) are selected and the mean estimate is considered as the final estimate [14, 24, 28, 33]. Using logit coefficient estimates ($\beta^{*}$) from pooled sample or from the appropriate reference group, the independent contribution of ${\text{X}}_{{\text{J}}}$ to the gap can be expressed as:

$$\frac{1}{{N^{2} }}\mathop \sum \limits_{i = 1}^{{N^{2} }} F\left( {X_{ji}^{1} \beta_{j}^{*} + \mathop \sum \limits_{J \ne j} X_{ji}^{1} \beta_{j}^{*} } \right) - F\left( {X_{ji}^{2} \beta_{j}^{*} + \mathop \sum \limits_{J \ne j} X_{ji}^{1} \beta_{j}^{*} } \right)$$

or

$$\frac{1}{{N^{2} }}\mathop \sum \limits_{i = 1}^{{N^{2} }} F\left( {X_{ji}^{2} \beta_{j}^{*} + \mathop \sum \limits_{J \ne j} X_{Ji}^{1} \beta_{J}^{*} } \right) - F\left( {X_{ji}^{2} \beta_{j}^{*} + \mathop \sum \limits_{J \ne j} X_{ji}^{2} \beta_{j}^{*} } \right)$$

One simpler strategy to overcome this issue involves using weights as well [22, 34, 35]. According to Yun [22], detailed decomposition using weights can be expressed as follows:

11 $$\overline{Y}^{1} - \overline{Y}^{2} = \sum\nolimits_{{{\text{k}} = 1}}^{{\text{k}}} {{\text{W}}_{{{\text{X}}_{{\text{k}}} }}^{\Delta } \left[ {\overline{{{\text{F}}\left( {x^{1} {\upbeta }^{1} } \right)}} - \overline{{{\text{F}}\left( {x^{2} {\upbeta }^{1} } \right)}} } \right] + \sum\nolimits_{{{\text{k}} = 1}}^{{\text{k}}} {{\text{W}}_{{{\upbeta }_{{\text{k}}} }}^{\Delta } \left[ {\overline{{{\text{F}}\left( {x^{2} {\upbeta }^{1} } \right)}} - \overline{{{\text{F}}\left( {x^{2} {\upbeta }^{2} } \right)}} } \right]} }$$

$${\text{If }}\sum\nolimits_{{{\text{k}} = 1}}^{{\text{k}}} {{\text{W}}_{{{\text{X}}_{{\text{k}}} }}^{\Delta } = } \sum\nolimits_{{{\text{k}} = 1}}^{{\text{k}}} {{\text{W}}_{{{\text{X}}_{{\text{k}}} }}^{\Delta } = 1}$$

where ${\text{W}}_{{{\text{X}}_{{\text{k}}} }}^{\Delta }$ and ${\text{W}}_{{{\upbeta }_{{\text{k}}} }}^{\Delta }$ represent the weight of *K*th variable in the linearization of the explained and unexplained components of inequality, respectively [22, 32]:

$${\text{W}}_{{{\text{X}}_{{\text{k}}} }}^{\Delta } = \frac{{{\upbeta }_{{\text{k}}}^{1} \left( {{\overline{\text{X}}}_{{\text{k}}}^{1} - {\overline{\text{X}}}_{{\text{k}}}^{2} } \right)}}{{\mathop \sum \nolimits_{{{\text{k}} = 1}}^{{\text{k}}} {\upbeta }_{{\text{k}}}^{1} \left( {{\overline{\text{X}}}_{{\text{k}}}^{1} - {\overline{\text{X}}}_{{\text{k}}}^{2} } \right)}}$$

$${\text{W}}_{{{\upbeta }_{{\text{k}}} }}^{\Delta } = \frac{{{\overline{\text{X}}}_{{\text{k}}}^{1} \left( {{\upbeta }_{{\text{k}}}^{1} - {\upbeta }_{{\text{k}}}^{2} } \right)}}{{\mathop \sum \nolimits_{{{\text{k}} = 1}}^{{\text{k}}} {\overline{\text{X}}}_{{\text{k}}}^{1} \left( {{\upbeta }_{{\text{k}}}^{1} - {\upbeta }_{{\text{k}}}^{2} } \right)}}$$

The Fairlie method mainly focuses on the explained portion of inequality without calculating the contribution of the differential effect from each factor to the unexplained part [14]. Nonetheless, that can be achieved through the practical technique proposed by Power et al. [32].

### Implementation of the decomposition in related Software

The Oaxaca command package is available for Stata [9], R [36] and SAS Macro% BO_decomp [37] to perform the blinder-oaxaca decomposition. In addition, Stata provides several packages developed for implementation of various forms of Blinder-Oaxaca decomposition into non-linear models, including fairlie [38], gdecomp [39], mvdcmp [32], and nldecompose [40] (Table 1).

**Table 1:** Different Stata command packages for decomposition of outcome differences between the two groups

Command	Description (estimation_command)
Oaxaca	Linear decomposition; the default (linear), logit decomposition (logit), probit decomposition (probit)
Fairlie	Logit model; the default (logit), probit model (probit)
Gdecomp	Logit model; the default (logit), probit model (probit), Poisson regression (poisson), negative binomial regression (nbreg)
Mvdcmp	Probit model (probit), Poisson regression (poisson), negative binomial regression (nbreg), complementary log–log model (cloglog)
Nldecompose	Linear regression (regress), logit model (logit), probit model (probit), Ordinal logit model (ologit), Ordinal probit model (probit), Tobit model (tobit), Interval regression (intreg), Truncated Gaussian regression (truncreg), Poisson regression (poisson), negative binomial regression (nbreg), *zero-inflated Poisson (*zip*),* zero inflated negative binomial (zinb), *Zero*-*truncated* poisson (ztp), *Zero*-*truncated* negative binomial (ztnb)

## Conclusions

### Drawbacks of the B-O decomposition models

The B-O decomposition methods have been subject to some criticisms that mostly focus on the model specification and the selection of the explanatory variables for the model [11, 30, 41].

The decomposition does not consider the different distribution of outcome among the individuals of each group [3]. It provides only information about the difference in mean predicted outcome between the 2 groups which is different from crude difference to the extent that distribution of other covariates between two groups are different.

The decomposition estimates also vary depending on the choice of reference group. There is often no compelling reason to choose the best group.

The 2 groups are not comparable due to unknown factors, putting the estimates subject to the effect of selection bias. In addition, the measurement errors that are systematically different for the groups can distort the results. Thus, there will be inefficiencies in the estimation of coefficients and consequently the "unexplained” component [3]. Additionally, if there is some misspecification in the outcome model and the average of residuals of the outcome model is not zero, there will be also another unexplained part which is the difference between among difference of mean observed outcomes and difference of mean predicted outcomes.

As we mentioned before, the unexplained part is sometimes referred to as the discrimination part. But for this part to be the exact discriminatory part, all the determinant of the outcome should exist in the model, otherwise this discriminatory part may be over or under estimated. Omitted variables or information bias can be some caused of this over/under estimation [15].

### Application of B-O method in health issues

This B-O decomposition method can be applied to explain inequalities in health outcome across any two groups, which defined based on race, gender, socioeconomic status, and so on. Using the method, the inequality can be decomposed into two general components; The first component is explained by the differences in the level (distribution or average) of observed related factors or characteristics between 2 comparison groups. The second represents the rest of inequality that not explained by such differences. In fact, existence of inequality despite identical individual characteristics can be rooted in unknown or un-measurable factors that affect the health outcomes. It may also result from “differential effect” of the observed characteristics (group difference in the magnitude of regression coefficients) across comparison groups. Each of these components can be decomposed into smaller components depending on the number of characteristics (variables) with the potential to create inequality (detailed decomposition).

Statistically, the “differential effect” in the decomposition model arises from the interaction effect of the related factors with the group’s indicator and can be interpreted in two ways; one depends on the nature of the variable itself, referring to the "behavioral response" of the explanatory variables in the two comparison groups such as different health behaviors and/or different individual tendency toward that behavior. One instance is the likelihood of smoking or deciding to start smoking in different communities or different social groups. Such difference can be due to cultural, environmental or attitudinal disparities in those communities [42]. The other is affected from the outside due to discrimination between the two groups in terms of associated factors (characteristics) such as unequal accessibility to health care services, and different quality of education, which in turn leads to different outcomes in the two groups. For example, if we assume that education level is the only known factor contributing to health behavior, the group difference in health behavior, despite the same level of education, can be due to varying qualities of education. This can be attributed to “differential effect” of education on health behavior in the absence of information on the quality of education.

Therefore, in addition to individual and social factors contributing to health, there are unequal or even unfair macro policies, social and economic programs playing a crucial role [5]. Assuming that individual and social characteristics remain the same in different subgroups of the population, inequality is expected to persist because of different government policies and programs. This implies that groups with specific characteristics receive different health programs and even at different quality. Hence, the conditions for inequality are set by physical, cultural, social and economic status of a community, giving rise to different, and in some cases, unfair opportunities.

In summary, the above decomposition methods can be applied in the health sciences in an effort to identify the contribution of each unevenly distributed factor as well as their different effects to the gap. It can therefore be indicated to what extent the average outcome varies according to changes in each factor while assuming the other factors are constant. Moreover, it will determine the overall share of unknown factors in creating inequality. In fact, the residual difference will be estimated while assuming that the distribution of observed factors remains identical [43].

It concludes that the application of the decomposition methods in the health inequality can identify the relative contribution of each particular factor in moderating the current inequality. Therefore, more detailed information can be provided for government planners and policy-makers, especially concerning modifiable factors [23, 44].

### Applied example

We illustrate the Blinder- Oaxaca Decomposition model using the available data from the 2011 STEPS Non-communicable Disease Risk Factors Survey of Iran. The survey was a population-based study according to STEPwise approach to the WHO non-communicable disease risk factor surveillance [45, 46].

The primary outcome was risk of obesity and overweight between urban and rural adults (residency: 0 = Urban, 1 = Rural). A set of variables including age (agey), gender (1 male, 2 female), socioeconomic stutus (SES) and physical activity (metmwcat: at least 600 MET-minutes per week) were considered as the predictors. The decomposition analysis was conducted in the Stata statistical software (v.14) using an updated Oaxaca package described by Yun [22]. The package included methods to handle the path dependency and identification problems [28–30]. The analysis has been performed for adults aged 15–69 years.

### The threefold (interaction) decomposition type

We decompose predicted rural–urban difference in Body Mass Index (bmi) using the Blinder-Oaxaca decomposition for linear models. The overall and detailed results are presented  in output 3.

“oaxaca” command in Stata computes the threefold decomposition from the perspective of Group 2 (Eq. 4), unless “threefold(reverse)”, “weight()” or “pooled” is specified. In this example, “threefold(reverse)” option expresses the threefold decomposition from the perspective of Group 1 (Eq. 5). That means group 2 (i.e., rural adults with a low average BMI) are selected as the reference for analysis. As discussed in the text, for nominal predictors such as gender, the detailed decomposition estimates depend on the choice of the base (omitted) category (Identification Problem). A solution is to perform the decomposition based on "normalized" effects (gender:normalize(gender?)) which recognizes sets of dummy variables representing nominal predictor and converts the coefficients so that the results are constant to the choice of the baseline [30].

In our sample, the mean predicted BMI is 26.4 for urban adults and 25.24 for rural adults, yielding a BMI disparity of 1.156. In general, only about 17.5% (0.202/1.156) of the disparity was due to the different distribution of the predictors (endowments). Among them, SES contributed the most (0.158/1.156 = 13.72%). In other words, reducing the difference of SES between the rural and urban adults will lead to a reduction of approximately 14% in the disparity. Furthermore, about 25% (0.43/1.24) of the disparity was attributed to the differential effect of the covariate entered in the model (coefficients effect) including general effect of unknown factors (_cons). This component specifies the unexplained portion of the disparity. The differential effect of age (0.916/1.156 = 79.2%) had the greatest contribution to this part of the disparity, followed by physical activity (metmwcat) and SES. The negative contribution of SES implies that removing the rural/urban difference in SES widens the disparity. Moreover, the ‘interaction part’ refers to the gap that is explained by the interaction between the endowment and coefficient effects.

Similarly, the predicted rural–urban difference in prevalence of overweight and obesity (bmicat) has been decomposed using B-O decomposition for non-linear models. The results are presented in Output 1. As shown, the prevalence remained significantly higher among urban (57.34%) compared to urban adults (46.89%) controlling for age, SES, gender and physical activity. The decomposition results indicate that different level of SES and differential effect of age had the greatest contribution to the difference as well.

**Output 1:**
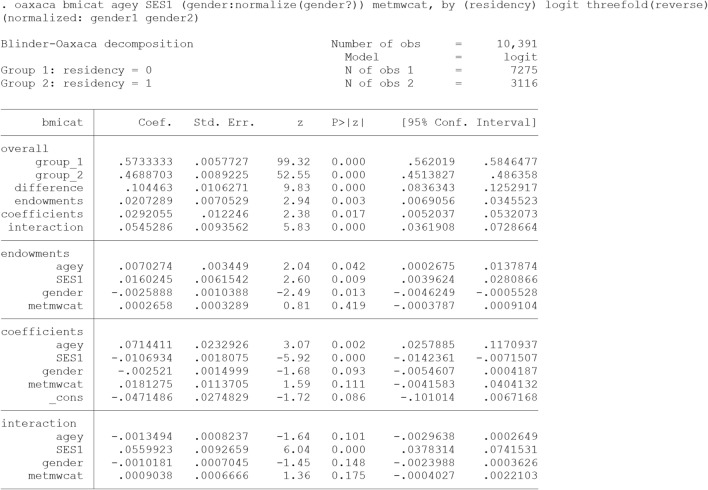
Threefold (interaction) Blinder-Oaxaca decomposition for non-linear models using rural adults (group 2) as the reference (from perspective of Group 1)

The “oaxaca” command in Stata also supports the non-linear decomposition for binary outcome. “logit” causes the non-linear decomposition for a binary outcome to be computed using the weighting method described by Yun [22].

The threefold decomposition results indicate that the mean predicted BMI is generally higher in urban than in rural adults and that the prevalence of obesity and overweight is consequently higher in urban adults. This is attributed to an obesogenic environment that promotes obesity-related behaviours such as unhealthy diet and insufficient physical activity. Consistent with this findings, in many developing countries urbanization and its related lifestyle changes, are considered significant risk factors for obesity and overweight. However, it was not the case in the study of Trivedi et al. [47].

Persistent of the disparity after adjusting for some obesity-related factors call for further investigation in this issue, which suggests that much of the difference between urban and rural residents is also driven by other unknown factors. Moreover, the findings suggest that the effect of age on obesity and overweight risk is different across the both rural and urban adults. To be more precise, the urban adults experienced higher obesity risk with increasing age than rural ones. This is in line with WHO report discussing that in developing countries, rural adults still maintaining a classic lifestyle gained little weight with age [48]. Accordingly, effective programs are needed to help elderly urban adults reduce high risks for obesity and unhealthy lifestyles.

As a general conclusion, obesity risk reduction policies need to consider not only rural/urban adults but also how it interacts with associated factors that make some subgroups more vulnerable than others. Generally, the application of the decomposition methods in the health inequality can identify the relative contribution of each particular factor in moderating the inequality. Therefore, more detailed information can be provided for government planners and policy-makers, especially concerning modifiable factors [23, 44].

### The twofold (discrimination) decomposition type

An alternative decomposition commonly used in the discrimination literature is the twofold decompositin (Eqs. 6, 7 and 8). In “Oaxaca” command in Stata, this decomposition can be performed, where “weight()” or “pooled” specifies the choice of the reference coefficients.

The results after using the “weight(0)” option are presented in output 2. It indicates that the coefficients from the group 2’s (rural adults’) model are used as the reference (non-discriminating). On the contrary, “weight(1)” specifies group 1 coefficients as the standard. In our case, the rural adults (Group 2) with a lower average of BMI was preferred as the reference.

As is evident from the output  2, the “unexplained” component is exactly similar to the “coefficients” component of the three-fold decomposition (Output 3). This component is often used as a measure for discrimination, but it also subsumes the effects of group differences in unobserved predictors. For this part to be the exact discriminatory part, all the determinant of the outcome should exist in the model, otherwise this part may be over or under estimated.

**Output 2:**
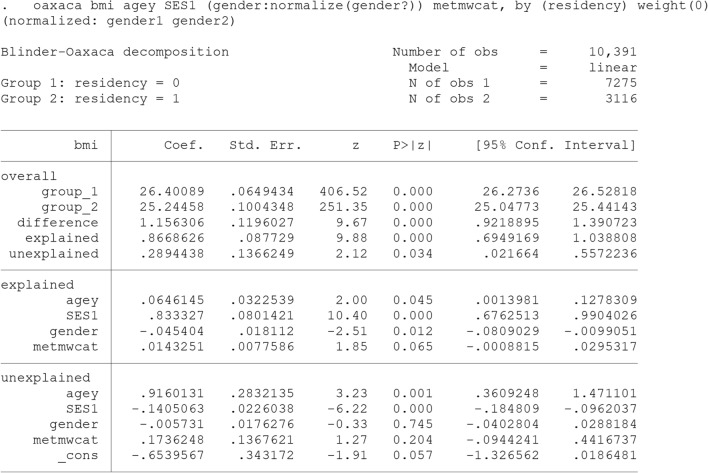
Twofold (Discrimination) Blinder-Oaxaca decomposition for linear models using the coefficients from rural adults’ model as the reference coefficients

**Output 3:**
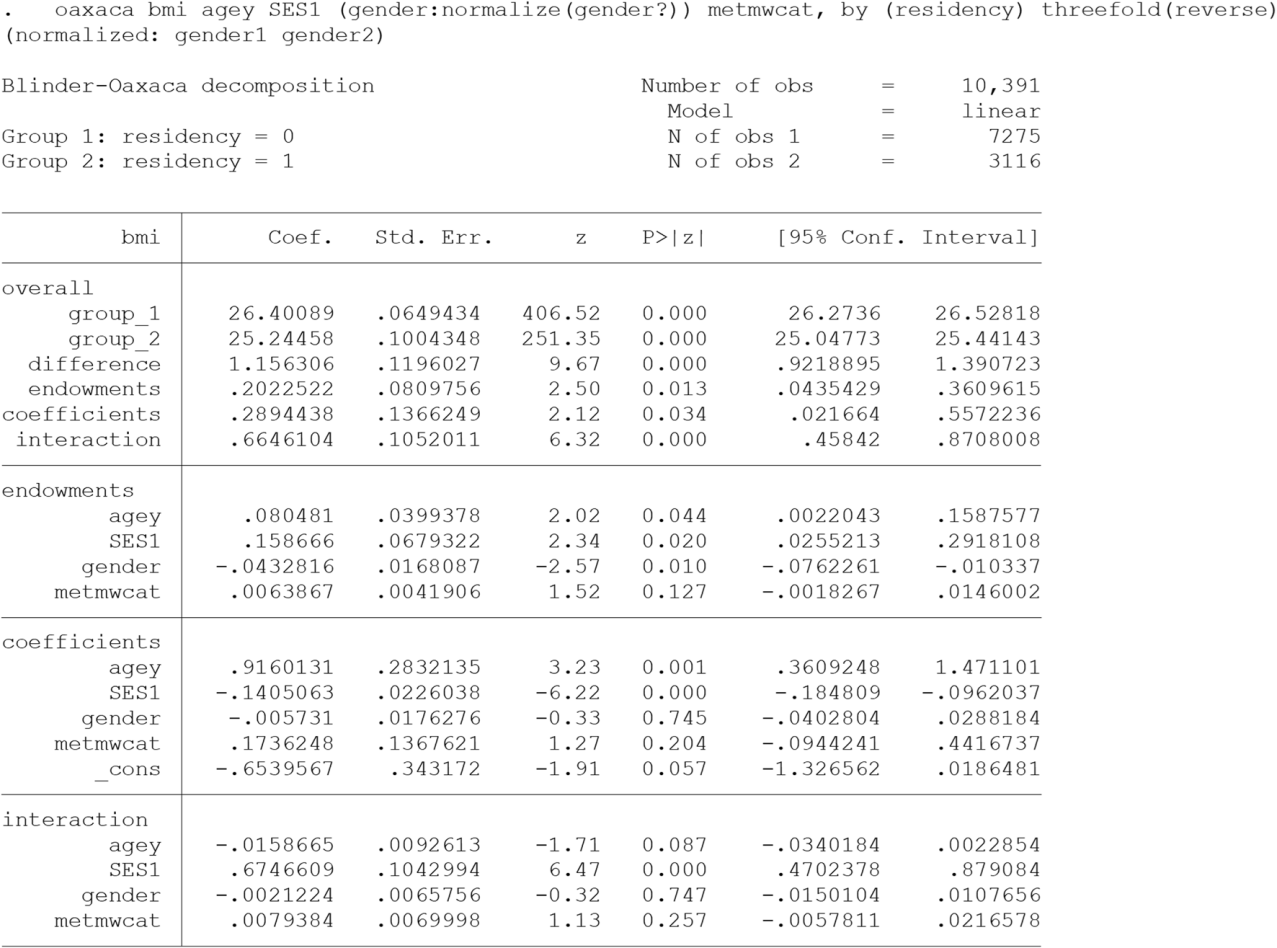
Threefold (interaction) Blinder-Oaxaca decomposition for linear models using rural adults (group 2) as the reference (from perspective of Group 1)

As shown, the differences in the level of observed covariates (the explained component) accounted for about 75% (0.867/1.156) of the total disparity. This component is the combination of “endowments” and “interaction” parts of the three-fold decomposition (Output 3). Although this component is called the explained component in two-fold decomposition in many texts, but some part of it (the interaction part) is in fact the simultaneous difference of coefficients and covariates level in both groups. Hence, if somebody wants the crude explained component, three folds’ decomposition can provide this crude explained part.

In the Output 4, “pooled” specifies that the coefficients from the pooled model over all cases be used for the decomposition. The results also indicate that different level of SES and differential effect of age had the greatest contribution to the difference. However, it is clearly shown that the decomposition estimates vary depending on the choice of reference group (index problem). There is often no compelling reason to choose the best group.

**Output 4:**
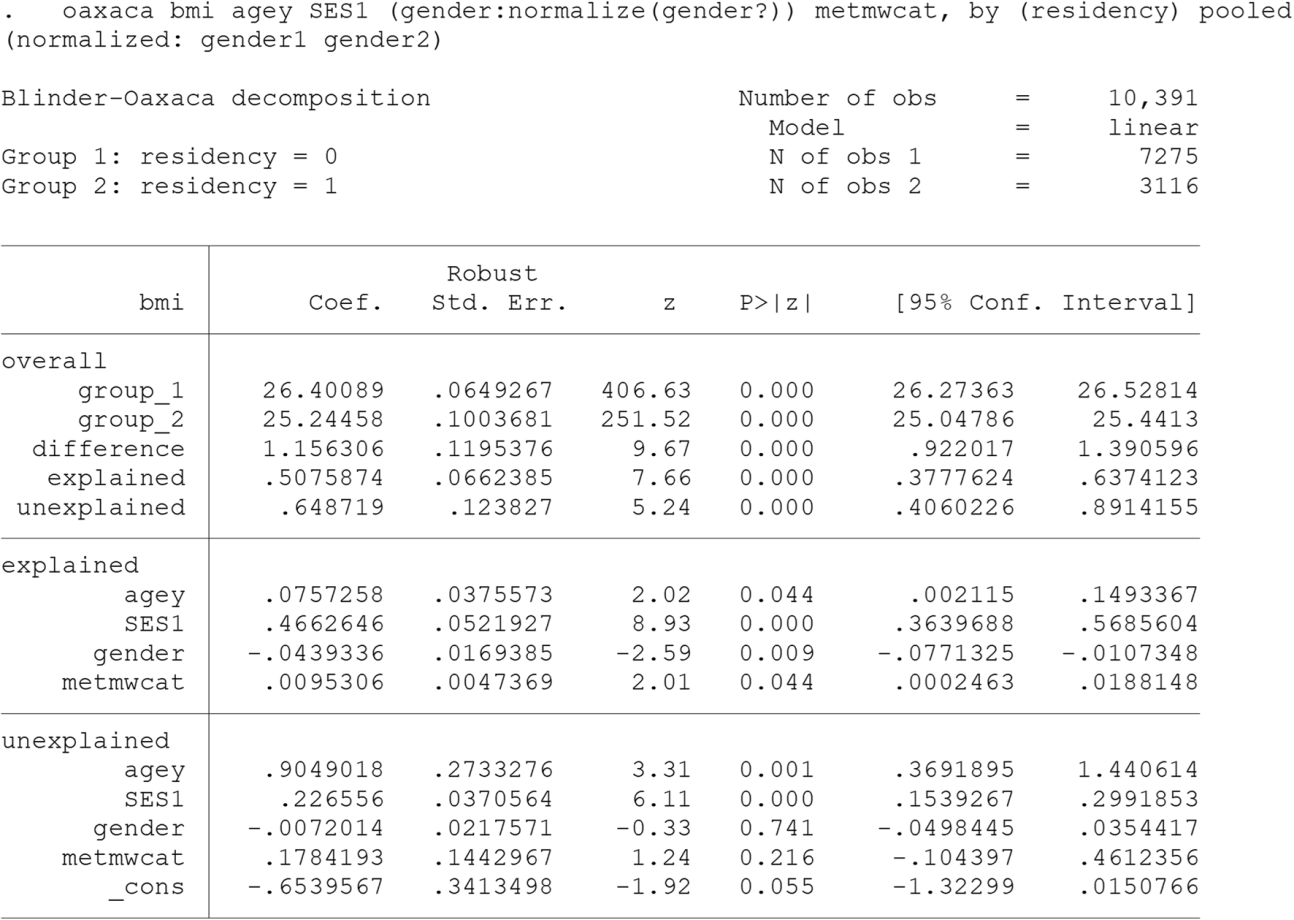
Twofold (Discrimination) Blinder-Oaxaca decomposition for linear models using the coefficients from pooled model over both groups as the reference coefficients

Alternatively, the twofold decomposition can be requested for non-linear models. In the Output 5, the predicted rural–urban difference in the prevalence of overweight and obesity (bmicat) has been decomposed using Blinder-Oaxaca decomposition for non-linear models.

**Output 5:**
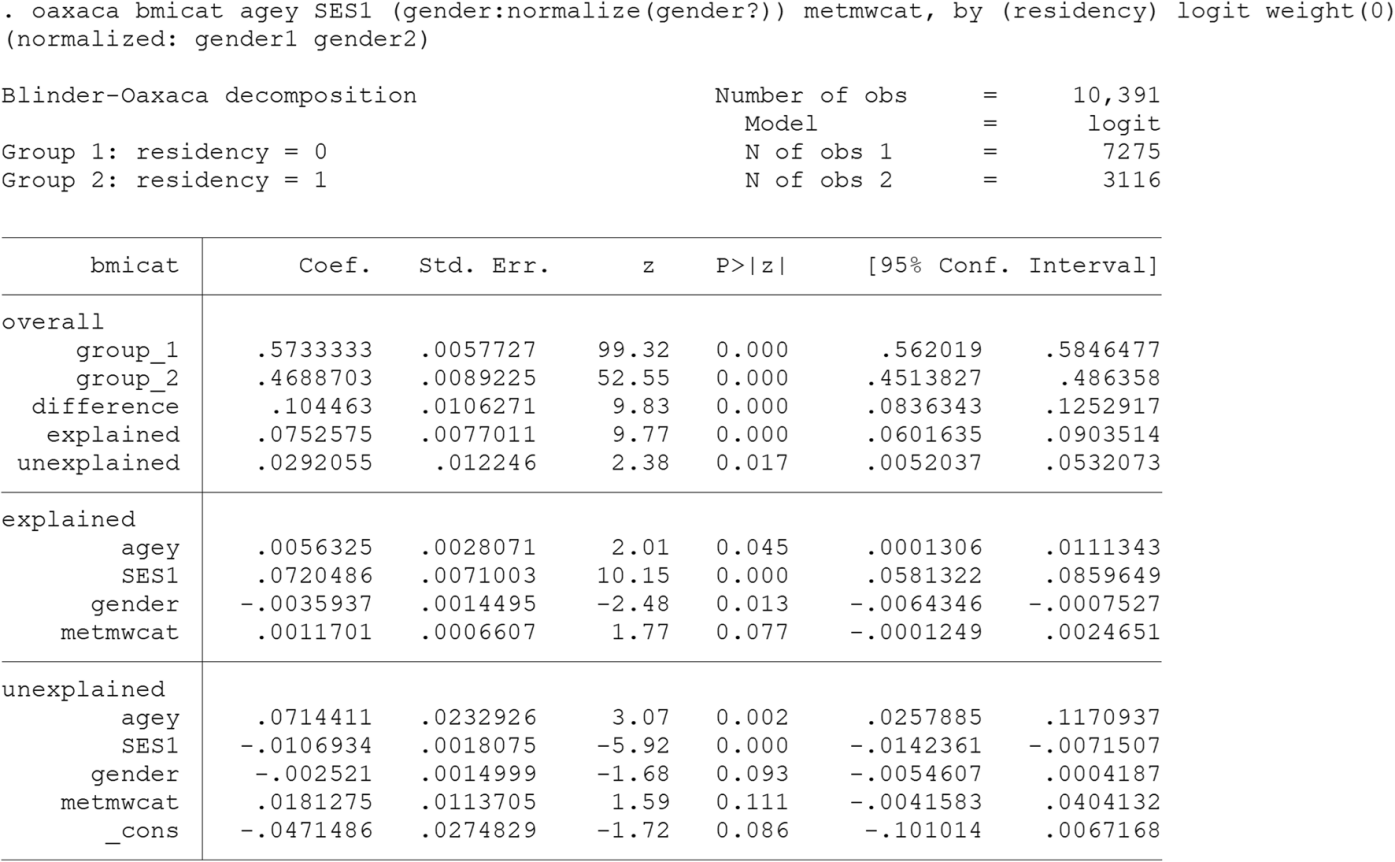
Twofold (Discrimination) Blinder-Oaxaca decomposition for non-linear models using the coefficients from rural adults’ model as the reference coefficients

An alternative non-linear decomposition command for binary outcome is available as “fairlie” [14].

The primary outcome is difference in the proportion of obesity and overweight (bmicat) between the rural and urban adults (residency: must be coded as 0 and 1). Accordingly, the technique decompose the rural/urban difference in "mean predicted probability of outcome". However, it mainly focuses on the explained portion of inequality without calculating the contribution of the differential effect from each factor to the unexplained part [14].

The main concern with the non-linear model is sensitive to the order of variables being included into the decomposition process (path dependency). The fairlie technique solving the problem by randomly ordering the variables across replications of the decomposition [28].

“ro” option causes the ordering of variables to be randomized in the analysis. reps(#) defines the number of decomposition replications. Thus, the multiple random sub-samples (e.g. 100 or 1000 times) of the majority group (equal to the sample size of minority group) are selected and the mean estimate is considered as the final estimate [14, 24, 28, 33].

ref(#) specifies the reference coefficients to be used with the decomposition. “ref(1)” indicates that the coefficients from the group =  = 1 model (rural adults’) are used. It is equivalent to the “weight (0)” in twofold Blinder-Oaxaca decomposition models.

Outputs 5 and 6 reports estimates from two decomposition methods, the non-linear Blinder-Oaxaca technique and the fairlie technique, for the rural/urban disparity in the prevalence of overweight and obesity. The prevalence also remained significantly higher among urban compared to urban adults controlling for age, SES, gender and physical activity. This is consistent with all the above reports. Approximately, 72% (0.0753/0.104) of disparity explained by rural/urban differences in these predictors. Among them, SES contributed the most.

**Output 6:**
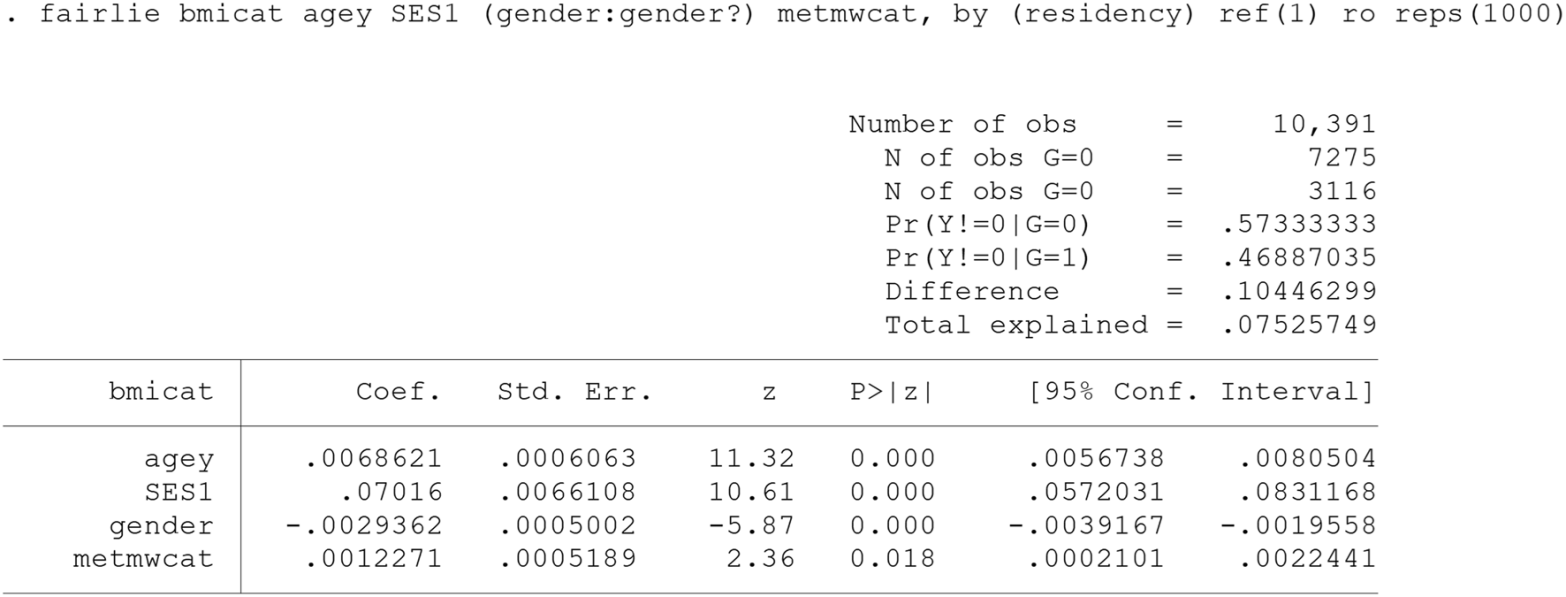
Fairlie decomposition model using the coefficients from rural adults’ model as the reference coefficients

## Data Availability

Not applicable.

## Abbreviations

B-O: Blinder-Oaxaca decomposition method
Uc: Unexplained Component
Ec: Explained Component
BMI: Body Mass Index
SES: Socioeconomic Status
